# Study of General and Pathological Anatomy

**Published:** 1835

**Authors:** 


VI.—Study of General and Pathological Anatomy.

As far as we know, the first chair of *General and Pathological Anatomy* instituted in the United States, is that of the new medical department of the Cincinnati College, which was announced in our last number. Dr. Gross, the professor, a gentleman whose course of studies and mental temperament, peculiarly qualify him for the duties of such a chair, has already made a valuable collection of morbid specimens, which he is augmenting daily. They will be arranged in the College edifice, and at all times open to the inspection of the members of the profession; from whom, in turn, the Doctor will be gratified to receive such specimens of a similar kind, as occasional *post mortem* examinations may put in their possession. They should be accompanied with an accou[illegible] the symptoms under which the patients, from whom t[illegible]

were taken, labored. To fit a specimen for transportation, it should be thoroughly washed with cold water, and then corked or sealed up, in a wide mouthed bottle, entirely full of strong whiskey or alcohol. Dr. G. will also be thankful for specimens taken from brute animals, which should also be accompanied with notices of the fatal symptoms.

In his teachings of General Anatomy and Physiology, the Professor intends to dip into the Comparative, and is desirous of enlarging his collection of Comparative Osteology—especially of acquiring the skeletons of our indigenous animals. In consequence of the death of a large lion, in one of the traveling menageries, which visited our city last year, he has obtained the bones, and several of the soft parts of that animal; and being desirous of comparing the former with bones of our American panther, would be glad to procure them.

